# Association between food insecurity and access to a mental health professional: cross-sectional analysis of NHANES 2007–2014

**DOI:** 10.1186/s12889-021-10818-5

**Published:** 2021-04-19

**Authors:** Nina Camille Burruss, Marina Girgis, Karen Elizabeth Green, Lingyi Lu, Deepak Palakshappa

**Affiliations:** 1 Wake Forest School of Medicine, Winston-Salem, NC USA; 2 Present address: Department of Psychiatry, University of North Carolina Hospitals, Chapel Hill, NC USA; 3 Departments of Internal Medicine and Pediatrics, Vanderbilt School of Medicine, Nashville, TN USA; 4 Department of Psychiatry and Behavioral Medicine, Wake Forest School of Medicine, Winston-Salem, NC USA; 5 Division of Public Health Sciences, Wake Forest School of Medicine, Winston-Salem, NC USA; 6 Departments of Internal Medicine and Pediatrics, Wake Forest School of Medicine, Medical Center Blvd, Winston-Salem, NC 27157 USA

**Keywords:** Food insecurity, Mental health professional, Depression, Social determinants of health, NHANES

## Abstract

**Background:**

To determine if individuals with food insecurity (FI) were less likely to have seen a mental health professional (MHP) within the past year than individuals without FI.

**Methods:**

This is a cross-sectional analysis of data from the National Health and Nutrition Examination Survey (NHANES) conducted in the United States between 2007 and 2014. All participants 20 years of age or older were eligible for this study. We excluded participants who were pregnant, missing FI data, or missing data from the Patient Health Questionnaire (PHQ-9). The primary outcome was self-reported contact with a MHP in the past 12 months. We used multivariable logistic regression models to test the association between FI and contact with a MHP, controlling for all demographic and clinical covariates.

**Results:**

Of the 19,789 participants, 13.9% were food insecure and 8.1% had major depressive disorder (MDD). In bivariate analysis, participants with FI were significantly more likely to have MDD (5.3% vs 2.8%, *p* < 0.0001) and to have been seen by a MHP in the preceding 12 months (14.0% vs 6.9%, *p* < 0.0001). In multivariable models, adults with FI had higher odds of having seen a MHP (OR = 1.32, CI: 1.07, 1.64).

**Conclusions:**

This study demonstrates that individuals with FI were significantly more likely to have seen a MHP in the preceding 12 months compared to individuals without FI. Given the growing interest in addressing unmet social needs in healthcare settings, this data suggests that visits with MHPs may be a valuable opportunity to screen for and intervene on FI.

## Background

Food insecurity (FI), defined by the United States Department of Agriculture (USDA) as inconsistent access to food due to social or economic constraints that limit intake and nutrition, is a major public health problem in the United States (U.S.) [1]. Prior to the COVID-19 pandemic, 10.5% of U.S. households, or over 35 million Americans, were food insecure at some point during 2019 [2]. Although the exact number is still unknown, it is estimated that an additional 17 million people in the U.S. will likely be food insecure due to the economic impact of the COVID-19 pandemic. Various factors including, but not limited to, mental health, income, employment, and race/ethnicity, are risk factors that have been shown to impact individuals’ and households’ access to food [3–6].

Individuals living in a food insecure household are at higher risk of worse health outcomes, and there is a well-documented relationship between FI and mental health conditions, particularly depression [3–6]. The association between FI and depressive symptoms is thought to be bidirectional [7]. Individuals with mental illnesses, such as major depressive disorder (MDD), may be more likely to experience FI because they are less likely to work or to access food and other supportive resources due to their depressive symptoms. Conversely, FI is thought to be an independent risk factor for the development of mental illness even when controlling for socioeconomic status [6]. Because of the increased psychological stressors associated with difficulty securing adequate nutrition, adults who are food insecure may develop mental illness, and there is a positive correlation between worsening FI and the number of reported depressive symptoms [5, 8].

Additionally, FI has also been associated with worse patterns of healthcare use, including delays in seeking necessary medical care. Individuals in food insecure households are less likely to report a usual source of care and more likely to postpone necessary preventative medical care due to competing demands; for example, individuals have to choose between spending money on food versus medical care [9, 10]. FI has also been associated with increased acute care utilization and total healthcare expenditures [10, 11]. Additionally, multiple studies have shown that individuals with MDD, regardless of food security status, are more likely to interact with healthcare professionals than individuals without MDD [9, 12]. The relationships between FI, preventative care, and acute care have been established, however, there is less information regarding the association between FI and mental healthcare utilization [13].

Although the relationship between FI and MDD has been established, there is limited data on the association between FI and access to a mental health professional (MHP). To fill this gap in the literature, our primary goal was to determine if individuals living in a food insecure household were more likely to have seen a MHP in the prior 12 months than individuals who did not live in a food insecure household.

## Methods

### Study design and data sources

We conducted a pooled cross-sectional analysis of the 2007–2014 National Health and Nutrition Examination Survey (NHANES). The National Center for Health Statistics conducts NHANES, which is a series of large, cross-sectional surveys. Participants in NHANES undergo an in-home interview on a variety of demographic and health characteristics, a physical examination conducted at a mobile examination center, and laboratory measurements. Results of NHANES are weighted to represent the non-institutionalized U.S. population. Further details of the study design and procedures have been previously published [14]. For this study, we combined data from the 2007–2008, 2009–2010, 2011–2012, and 2013–2014 waves of NHANES.

### Study sample

All participants who were 20 years of age or older were eligible to be included in this study (unweighted *N* = 23,482). We excluded participants who were pregnant (*N* = 247), missing FI data (*N* = 228), or missing data on the patient health questionnaire (PHQ-9; *N* = 3218) for a total unweighted sample size of 19,789. The total weighted sample size used for this study was 608,437,356.

### Outcome

Our primary outcome was contact with a MHP in the past 12 months (yes or no). All respondents are asked “During the past 12 months, have you seen or talked to a mental health professional such as a psychologist, psychiatrist, psychiatric nurse, or clinical social worker about your health?” and our outcome was based on self-report to this question.

### Exposure

Our exposure of interest was household FI. FI was measured in NHANES using the 18-item Food Security Survey Module [15]. This validated questionnaire was developed by the USDA to measure household FI over the prior 12 months. NHANES administers the Food Security Survey Module in accordance with the USDA recommendations and uses the established scoring system for responses to the items. Households are categorized as having high food security (0 affirmative response), marginal food security (1–2 affirmative responses), low food security (3–7 affirmative responses), and very low food security (≥ 8 affirmative responses). Consistent with USDA definitions, we categorized participants with low or very low food security as food insecure, and we analyzed FI as a binary variable for this study (food secure: 0–2 affirmative responses; food insecure: ≥3 affirmative responses). Sensitivity analyses evaluating FI as a 4-category variable found similar results (data not shown).

### Covariates

Based on prior studies, our covariates included demographic and clinical characteristics that have been associated with FI and/or care by a MHP [2, 5, 16]. These included age, gender, race/ethnicity (non-hispanic White, non-hispanic Black, Hispanic, or other), and birthplace (U.S. or not). We also included marital status, highest educational level achieved (< high school, graduated high school, or > high school), household income-to-poverty ratio, health insurance (private, Medicare, public not including Medicare, or none), smoking status (current, former, or never), and if a participant reported using a nutrition assistance program (including Supplemental Nutrition Assistance Program (SNAP), Special Supplemental Nutrition Assistance Program for Women, Children, and Infants (WIC), or received food from a food pantry or soup kitchen).

We also included medical comorbidities including diabetes, dyslipidemia, hypertension, cardiovascular disease, and MDD. Consistent with prior studies using NHANES data [17–19], we categorized diabetes, dyslipidemia, and hypertension based on questionnaire, physical examination, and laboratory data. A participant was defined as having diabetes if they had any of the following: self-report of diabetes, glycated hemoglobin > 6.5%, fasting glucose > 126, or currently taking a diabetes medication (including sulfonylurea, insulin, or an incretin mimetic). Dyslipidemia was defined as having any of the following: self-report of high cholesterol, currently taking a cholesterol lowering medication, or laboratory diagnosis of dyslipidemia (total cholesterol ≥240 mg/dl, HDL < 30 mg/dl, LDL ≥160 mg/dl, Triglycerides ≥150 mg/dl). Hypertension was defined as any of the following: self-report of high blood pressure, currently taking a blood pressure medication, or elevated blood pressure based on the average of 3 blood pressure readings (Systolic Blood Pressure > 140 mmHg or Diastolic Blood pressure > 90 mmHg). Cardiovascular disease was determined by self-report of a history of coronary artery disease, angina, prior myocardial infarction, congestive heart failure, or stroke.

MDD was determined by responses to the PHQ-9 [20, 21]. The PHQ-9 consists of nine items that assess the frequency of depressive symptoms over the prior 2 weeks. Questions include how often over the last 2 weeks have you felt down or depressed and how often have you had thoughts that you would be better off dead or hurting yourself in some way. Responses include “not at all”, “several days”, “more than half the days”, and “nearly every day” and are given a point ranging from 0 to 3. We categorized participants as having MDD (yes or no) if they had a score of ≥10 on the PHQ-9 [20, 21].

### Statistical analysis

Participant characteristics were presented as means (standard deviation) for continuous variables or N (percent) for categorical variables. We performed bivariate analyses testing the association between FI, contact with a MHP, and all individual covariates using t-tests or chi-square test. We used multivariable logistic regression models to test the association between FI and contact with a MHP, controlling for all demographic and clinical covariates. A priori, we hypothesized MDD as a possible effect modifier. We did not find a significant interaction term, so we treated MDD as a confounder. We also evaluated for significant differences in the association between FI and contact with a MHP based on participant characteristics (age, gender, race/ethnicity, marital status, highest education level achieved, birthplace, health insurance, and medical comorbidities). For participant characteristics with a potentially significant interaction (*p* < 0.2), we used predictive margins to determine the adjusted proportion who reported contact with a MHP. All analyses accounted for the complex survey design of NHANES by applying the appropriate sample weights, clustering, and the primary sampling unit. As only a small number of participants were missing data in variables (< 8%), we used listwise deletion to account for missing data. We used a two-sided hypothesis test and considered a *p*-value of < 0.05 statistically significant. All analyses were conducted using Stata 15.1 (StataCorp, College Station, TX). The Wake Forest School of Medicine Institutional Review Board deemed this study of publically available, de-identified data exempt from human subjects research.

## Results

### Study population characteristics

Of the 19,789 participants (weighted study population *N* = 608,437,356), the majority was female (50.7%), married (55.6%), and had greater than a high school education level (60.7%) (Table 1). The mean age was 47.5 years (SD = 16.8 years) and the mean household income-to-poverty ratio was 3.0 (SD = 1.7). We found that 8.1% of participants were identified as having MDD. Of the participants, 13.9% lived in a food insecure household with 20.6% reporting SNAP usage, 6.5% reporting WIC usage in the past year, and 7.1% reporting emergency food (food pantry, soup kitchen) usage.

**Table 1:** Study population characteristics

	*N* = 19,789 (Weighted *N* = 608,437,356)	Weighted %
**Age**	Mean = 47.5 (SD = 16.8)	
**Gender**	Female	50.7
	Male	49.3
**Race/ethnicity**	Non-Hispanic, White	69.0
	Non-Hispanic, Black	11.0
	Hispanic	13.5
	Other	6.6
**Marital status**	Married	55.6
	Widowed	5.6
	Divorced	10.7
	Separated	2.3
	Never married	18.4
	Living with partner	7.4
**Adult education level**	< High school	16.8
	High school graduate	22.5
	>High school	60.7
**Household Income-to-poverty ratio**	Mean = 3.0 (SD = 1.7)	
**Born outside the US**	Yes	16.4
**Insurance**	Private	49.9
	Medicare	13.7
	Public, not Medicare	17.2
	None	19.2
**Smoking**	Never	54.7
	Current	20.7
	Former	24.6
**SNAP**	Yes	20.6
**WIC**	Yes, received in last 12 months	6.5
**Emergency food (food pantry, soup kitchen)**	Yes	7.1
**Diabetes**	Yes	12.6
**Dyslipidemia**	Yes	55.5
**Hypertension**	Yes	38.0
**Cardiovascular Disease**	Yes	7.7
**MDD**	Yes	8.1
**Food insecurity**	Yes	13.9
**MHP**	Yes	7.9

*SD* standard deviation, *SNAP* Supplemental Nutrition Assistance Program, *WIC* The Special Supplemental Nutrition Program of Women, Infants, and Children, *MDD* Major Depressive Disorder, *MHP* mental health provider

### Bivariate analysis of food insecurity and covariates

In bivariate analysis, FI was associated with younger age (mean 41.4 years vs 48.5 years), lower educational attainment (38.4% vs 64.3% with >high school education), lower household income-to-poverty ratio (mean 1.4 vs mean 3.2), birthplace outside of the U.S. (23.9% vs 15.1%), and no health insurance (40.6% vs 15.8%) (all *p* < 0.0001) (Table 2). Of the study participants, individuals with FI were more likely to have diabetes (14.7% vs 12.2%, *p* = 0.002) and cardiovascular disease (9.0% vs 7.4%; *p* = 0.03). Food insecure participants were also more likely to have MDD (5.3% vs 2.8%, *p* < 0.0001). Participants who lived in a food insecure household were significantly more likely to have been seen by a MHP in the last 12 months (14.0% vs 6.9%, *p* < 0.0001).

**Table 2:** Characteristics of food secure compared to food insecure participants

		Food secure (%)	Food insecure (%)	*p*-value
**Total population**	*N* = 19,789 (weighted study population *N* = 608,437,356)	86.1	13.9	
**Age**	Mean (SD)	48.5 (16.8)	41.4 (14.9)	**< 0.0001**
**Gender**				**0.39**
	Male	49.4	48.5	
	Female	50.6	51.5	
**Race**				**< 0.0001**
	Non-Hispanic, White	72.2	48.8	
	Non-Hispanic, Black	9.7	18.6	
	Hispanic	11.4	26.6	
	Other	6.6	6.0	
**Marital status**				**< 0.0001**
	Married	58.6	36.7	
	Widowed	5.8	4.8	
	Divorced/separated	11.9	19.7	
	Never married	17.2	25.9	
	Living with partner	6.5	13.0	
**Education level**				**< 0.0001**
	< High school	14.1	34.1	
	High school graduate	21.7	27.5	
	>High school	64.3	38.4	
**Income-to-poverty ratio**	Mean (SD)	3.2 (1.6)	1.4 (1.1)	**< 0.0001**
**Born outside US**	Yes	15.1	23.9	**< 0.0001**
**Insurance**				**< 0.0001**
	Private	53.7	25.7	
	Medicare	14.7	7.7	
	Public, not Medicare	15.8	26.1	
	None	15.8	40.6	
**Smoking**				**< 0.0001**
	Never	56.5	43.6	
	Current	17.7	39.1	
	Former	25.8	17.3	
**SNAP**	Yes	4.8	16.7	**< 0.0001**
**WIC**	Yes	4.2	2.3	**< 0.0001**
**Emergency food**	Yes	3.4	30.2	**< 0.0001**
**Diabetes**	Yes	12.2	14.7	**0.002**
**Dyslipidemia**	Yes	55.9	53.5	0.10
**Hypertension**	Yes	38.5	35.0	**0.01**
**Cardiovascular Disease**	Yes	7.4	9.0	**0.03**
**MDD**	Yes	6.2	20.4	**< 0.0001**
**MHP**	Yes	6.9	14.0	**< 0.0001**

*SD* standard deviation, *SNAP* Supplemental Nutrition Assistance Program, *WIC* The Special Supplemental Nutrition Program of Women, Infants, and Children, *MDD* Major Depressive Disorder, *MHP* mental health provider

### Food insecurity and contact with a mental health professional

We used multivariable logistic regression models to test the association between FI and contact with a MHP, controlling for all demographic and clinical covariates. Using these models, adults with FI were significantly more likely to report having had contact with a MHP in the prior 12 months than individuals living in a food secure household (OR = 1.32, 95% CI: 1.07, 1.64; *p* = 0.009) (Table 3).

**Table 3:** Multivariable analysis: Association between food insecurity and contact with a mental health professional

Mental Health Professional		Odds Ratio	95% CI	*p*-value
Food security	Secure	Ref		
	Insecure	1.32	1.07, 1.63	**0.009**
MDD	No	ref		
	Yes	4.04	3.19, 5.11	**< 0.001**
Age		0.99	0.98, 0.99	**0.001**
Gender	Male	Ref		
	Female	1.15	0.98, 1.35	0.073
Race/ethnicity	White	Ref		
	Hispanic	0.89	0.73, 1.08	0.247
	Black	0.65	0.52, 0.80	**< 0.001**
	Other	0.71	0.53, 0.94	**0.016**
Marital status	Married	Ref		
	Widowed	1.06	0.75, 1.48	0.75
	Divorced/separated	1.73	1.35, 2.21	**< 0.001**
	Never married	1.47	1.17, 1.83	**0.001**
	Living with partner	1.12	0.88, 1.43	0.35
Education level	<High school	Ref		
	High school graduate	1.44	1.13, 1.83	**0.004**
	>High school	2.16	1.73, 2.69	**< 0.001**
Born outside US	No	Ref		
	Yes	0.77	0.59, 0.99	**0.041**
Insurance	Private	Ref		
	None	0.79	0.61, 1.02	0.08
	Medicare	1.36	0.90, 2.07	0.152
	Public, not Medicare	2.02	1.62, 2.52	**< 0.001**
Smoking	Never	Ref		
	Current	1.67	1.34, 2.08	**< 0.001**
	Former	1.41	1.12, 1.76	**0.003**
SNAP	No	Ref		
	Yes	1.36	1.10, 1.70	**0.005**
WIC	No	Ref		
	Yes	0.70	0.52, 0.94	**0.016**
Emergency food (food pantry, soup kitchen)	No	Ref		
	Yes	1.48	1.16, 1.88	**0.002**
Income-to-poverty ratio		1.04	0.97, 1.12	0.28
DM	No	Ref		
	Yes	0.78	0.63, 0.96	**0.021**
Dyslipidemia	No	Ref		
	Yes	0.88	0.75, 1.05	0.152
HTN	No	Ref		
	Yes	1.12	0.91, 1.39	0.284
Cardiovascular disease	No	Ref		
	Yes	1.12	0.82, 1.54	0.461

We used multivariable logistic regression models to test the association between FI and contact with a MHP, controlling for all demographic and clinical covariates. *CI* confidence interval, *MDD* major depressive disorder, *SNAP* Supplemental Nutrition Assistance Program, *WIC* The Special Supplemental Nutrition Program of Women, Infants, and Children, *DM* diabetes, *HTN* hypertension

We found a potential interaction (*p* < 0.2) between FI and health insurance, FI and diabetes, and FI and hypertension (Table 4). Among participants insured by Medicare, 7.32% (95% CI: 5.02, 9.62%) who were food secure reported contact with a MHP compared to 19.52% (95% CI: 10.87, 28.17%) with FI. FI was associated with an adjusted difference of 8.35% (95% CI: 2.69, 14.00%; *p* = 0.004) among participants insured by Medicare. Among participants with diabetes, 5.65% (95% CI: 4.48, 6.82%) who were food secure reported contact with a MHP compared to 10.21% (95% CI: 7.75, 12.67%) with FI, and FI was associated with an adjusted difference of 4.30% (95% CI: 1.85, 6.75%; *p* = 0.001) among participants with diabetes. Among participants with hypertension, FI was associated with an adjusted difference of 4.52% (95% CI: 1.27, 7.77%; *p* = 0.007).

**Table 4:** Differences in Contact with a Mental Health Professional by Participant Characteristics

		Predicted Proportion (95% CI)	Adjusted difference (95% CI)	***p***-value
**Insurance**				
**None**	Food secure	5.47% (4.34, 6.60%)	Ref	
	Food insecure	7.01% (5.18, 8.83%)	1.72% (−0.39, 3.84%)	0.11
**Private**	Food secure	6.77% (5.72, 7.82%)	Ref	
	Food insecure	7.62% (5.54, 9.70%)	0.86% (−1.41, 3.12%)	0.45
**Public, not Medicare**	Food secure	12.45% (10.72, 14.17%)	Ref	
	Food insecure	14.23% (11.00, 17.47%)	1.94% (−2.08, 5.96%)	0.34
**Medicare**	Food secure	7.32% (5.02, 9.62%)	Ref	
	Food insecure	19.52% (10.87, 28.17%)	8.35% (2.69, 14.00%)	**0.004**
**Diabetes**				
**No**	Food secure	7.90% (7.15, 8.66%)	Ref	
	Food insecure	9.51% (8.02, 10.10%)	1.62% (0.00, 3.23%)	0.05
**Yes**	Food secure	5.65% (4.48, 6.82%)	Ref	
	Food insecure	10.21% (7.75, 12.67%)	4.30% (1.85, 6.75%)	**0.001**
**Hypertension**				
**No**	Food secure	7.59% (6.76, 8.41%)	Ref	
	Food insecure	8.45% (6.82, 10.07%)	0.14% (−1.26, 1.55%)	0.84
**Yes**	Food secure	7.74% (6.49, 8.98%)	Ref	
	Food insecure	12.00% (9.54, 14.47%)	4.52% (1.27, 7.77%)	**0.007**

## Discussion

Using this nationally representative U.S. sample, we demonstrate that individuals living in a food insecure household are significantly more likely to have seen a MHP in the preceding 12 months than individuals who do not live in a food insecure household. With the growing interest in addressing patients’ unmet social needs as a routine part of clinical care, this data suggests that MHPs may be an important area to develop interventions to screen patients for FI and help assist patients with unmet food needs.

The social determinants of health, or the areas that people grow, work, live, and age, have a profound impact on morbidity and mortality, and the social determinants can lead to unmet health-related social needs, such as FI [22–24]. Additionally, the COVID-19 pandemic has highlighted and exacerbated many of the underlying inequities that already existed in the U.S., and the rates of FI have increased due to the economic impact of the COVID-19 pandemic. Historically, addressing patients’ unmet social needs has been the focus of public health and public policy communities, but an increasing number of national U.S. healthcare organizations have recommended that health systems screen and address FI and other unmet social needs in clinical care settings to improve patient care and population health [25–27]. With these national recommendations in the U.S., there has been a growing body of evidence that addressing FI in clinical care settings can improve patients’ access to food resources [28, 29]. These efforts have primarily focused on primary care specialties, including pediatrics, family medicine, and internal medicine, as areas to address patients’ unmet social needs [10, 12, 30]. However, these specialties are often overburdened with screening expectations during short clinical encounters. Studies have demonstrated low screening rates for unmet health-related social needs during these visits, indicating potentially missed opportunities for intervention [31]. Further, prior studies have shown that individuals in food insecure households are less likely to present for preventative care visits, and many patients and families suffering from FI may be missed if screening programs only focused on preventative care visits [10, 11]. Our results demonstrate that individuals living in food insecure households were significantly more likely to have contact with a MHP than those who were food secure in the prior 12 months, controlling for depression. This is consistent with a prior study in Canada that used national survey data to demonstrate that food insecure individuals were more likely to have contact with a designated MHP, such as a psychiatrist, and an increasing degree of FI correlated with increased odds of seeking mental healthcare treatment [13]. MHPs and mental health clinics may be an additional area to screen patients for FI with a high likelihood of identifying patients with unmet food needs. In this way, MHPs may help offset the significant screening burden placed on primary care physicians, while increasing identification of those with unmet social needs.

Although our results were consistent with a prior study in Canada, given the issues with healthcare access that individuals with FI can experience in the U.S., it was a little surprising that participants who reported FI in our study were more likely to report seeing a MHP in the prior 12 months. There are several potential reasons why we may have seen this association. First, prior studies have shown that patients with FI are more likely to have a concomitant psychiatric illness such as MDD, and individuals with psychiatric illness are more likely to see a MHP [5, 7, 8]. Although we did not find a significant interaction between FI and MDD in our study, it could be that individuals’ with worse mental health are more likely to be food insecure and could be why we found a significant association between FI and contact with a MHP. This could also be why we found a significant interaction between FI and if participants received Medicare insurance given that individuals with certain mental health illnesses are eligible to receive Medicare before the age of 65 years. Second, we found that the association between FI and contact with a MHP varied if participants reported having Medicare insurance, diabetes, or hypertension. As older individuals and individuals younger than 65 years of age with certain medical disabilities are eligible to receive Medicare, the association between FI and contact with a MHP may only be among food-insecure participants with other comorbidities who already receive medical treatment for other conditions. Third, the question in NHANES asking if participants saw a MHP in the prior 12 months did not assess where or in what context this visit occurred (e.g. office setting, emergency department, or hospital). Patients with FI have been found to have higher rates of acute healthcare utilization with more frequent emergency department visits and hospitalizations [9, 10]. Food-insecure participants may have seen a MHP during these acute visits rather than in an ambulatory setting. Further research is needed to confirm our findings, though, and determine the potential reasons why food insecure individuals may be more likely to report seeing a MHP.

FI is an issue that affects a large proportion of households in the U.S. and is linked to depression, anxiety, and other mental health problems [5, 6, 32, 33]. Mental health problems may actually be one of the primary pathways through which FI leads to worse physical health outcomes, such as cardiovascular disease and cardiovascular risk factors [34]. As a growing number of national healthcare organizations are interested in mitigating the negative health effects of FI and other unmet social needs, understanding the complex relationship between FI and health could further tailor interventions for clinical care settings [25–27]. Although healthcare-based FI interventions have shown success in connecting patients to resources in the community, there is still limited data on if these interventions ultimately improve health outcomes [29]. These interventions have primarily focused on providing food resources to individuals with FI [28, 29], perhaps future interventions could focus simultaneously on lack of food and poor mental health to address FI and the accompanying sequelae.

MHPs have historically produced a large body of research on how social determinants of health affect the development and treatment of mental illness. However, expanding the role of the MHP to assess unmet social needs is an emerging area in the literature. Shields-Zeeman, Lewis and Gottlieb, describe an approach for directly addressing social determinants of health within the field of Psychiatry [35]. First, MHPs routinely elicit extensive social histories, so the evaluation of FI could be easily integrated into the psychiatric or psychological exam in a standardized manner. Brief 1 or 2-item FI questionnaires are available and have been validated in clinical care settings [36]. Second, MHPs often work in multidisciplinary settings alongside case managers, social workers, and other health professionals who are equipped to connect patients with health system, community, or government resources. Future studies could focus on developing and testing tools to address FI in mental health clinics.

Previous studies have suggested that providers may be less inclined to screen for social determinants of health if they are not aware of resources or perceive the resources available as insufficient [31]. In order to support FI screening by MHPs, it is critical that MHPs are informed of the government and community resources available to individuals with unmet social needs or are connected with social workers or other members of the care team who are familiar with the resources to which patients should be referred. In the U.S., this includes knowledge of SNAP, WIC, and local emergency food sources.

There are several limitations to this study that should be acknowledged. First, this study was cross-sectional, so we are unable to determine causation. Whether individuals who are food insecure are more likely to have been seen by a MHP or individuals who have been seen by a MHP are more likely to report FI is unclear. In either case, mental health clinics may be missing an opportunity to identify and address patients’ unmet food needs. Second, this study extrapolates the diagnosis of MDD from responses to the PHQ-9, which is intended to be a screening rather than a diagnostic tool. Areas for future study include assessing the association between FI and other mental illnesses such as schizophrenia or bipolar disorder, which may have a more prominent impact on food security. Third, having been seen by a MHP was based on self-report. Also, the question used to assess if a participant had seen a MHP asked if participants had seen a psychiatrist, psychologist, psychiatric nurse, or clinical social worker, rather than evaluating each provider individually. Future studies should consider evaluating more direct measures of mental healthcare use and provider type, such as administrative data. Fourth, a priori we hypothesized that depression would be a potential effect modifier between FI and contact with a MHP. We did not find a significant interaction term, so we treated MDD as a confounder. Future studies should evaluate if the relationship between FI and mental healthcare utilization varies by depression severity or mental health diagnosis.

## Conclusion

FI and depression are prevalent conditions that often occur concomitantly. In this nationally representative sample of adults in the U.S., we found that individuals with FI were significantly more likely to have had contact with a MHP in the prior year than individuals without FI, controlling for depression. Given the detailed social histories that MHPs elicit, the extended clinic visits they conduct, and the close relationships they form with patients, MHPs may be an important area to expand clinical social prescribing programs to assess and address patients’ unmet food needs.

## Data Availability

This study utilized publically available data from the National Health and Nutrition Examination Survey. This data can be accessed using the following link: https://www.cdc.gov/nchs/nhanes/index.htm
